# Geotrichosis Presenting As Funguria and Asymptomatic Urinary Tract Infection in a Patient with Renal Cyst

**DOI:** 10.7759/cureus.7616

**Published:** 2020-04-10

**Authors:** Venkataramana Kandi, Ritu Vaish, Padmajakshi Gurrapu, Sri Sandhya Koka, Mohan Rao Bhoomigari

**Affiliations:** 1 Clinical Microbiology, Prathima Institute of Medical Sciences, Karimnagar, IND; 2 Microbiology, Prathima Institute of Medical Sciences, Karimnagar, IND; 3 Microbiology, Rural Medical College, Pravara Institute of Medical Sciences (Deemed University)/Prathima Institute of Medical Sciences, Karimnagar, IND

**Keywords:** fungi, yeasts, saprophytes, infections in humans and animals, opportunistic infections, immunosuppressed individuals, geotrichum, geotrichosis, urinary tract infection, funguria

## Abstract

Fungi are a versatile group of microorganisms that exist in three morphological forms, which include the yeasts (oval/spherical budding cells), true fungi (produce long filamentous, branching structures called as hyphae/mycelia), and dimorphic fungi (show both yeast at 37^0^C and hyphal forms at room temperature). Most fungi are present in the environment and live as saprophytes. Some fungal species like the *Candida *are present in the human respiratory, intestinal, and genitourinary tract as commensals. Some fungi cause infections in humans and animals (dermatophytes). Few other fungal species are responsible for opportunistic infections, mostly in debilitated and immunosuppressed individuals. *Geotrichum* is one such fungus, which is present in the soil, dead, and decomposing organic matter, and may contaminate food, fruits, and vegetables. Geotrichosis is the infection caused by *Geotrichum *species. Due to its similarity in morphology, clinical features, and the pathogenicity with common fungi like the *Candida *species, and others, its clinical significance is undermined. This report presents a case of funguria and asymptomatic urinary tract infection caused by *Geotrichum *species in a patient with a renal cyst.

## Introduction

*Geotrichum *is a yeast-like fungus, which is ubiquitous and present in the environment as a saprophyte. *Geotrichum *may contaminate the food, fruits, and vegetables and in turn, cause a human infection called geotrichosis, a rare opportunistic mycotic infection. *Geotrichum *is also called as a machinery mold because of its ability to grow on various surfaces like the walls of the equipment, moist building walls, floors, and gutters. They adhere to the surfaces and produce slimy films. There is a debate about the categorization of *Geotrichum *as a yeast because of the appearance of long filamentous and branching structures which break into arthroconidia, as well as the characteristic growth of the colonies on agar surface (pit within the agar as mycelia penetrate under the surface). Some *Geotrichum *species (spp.) are also used in the preparation of cheese [1].

Although a saprophyte, *Geotrichum *spp. are found as commensals in the mouth, skin, respiratory tract (upper, and lower), gastrointestinal tract, and genitourinary tract of humans and animals [2]. Opportunistic infections with *Geotrichum *spp. were reported previously among patients suffering from leukemia, neutropenia, and renal transplant patients [3,4]. Urinary tract infection (UTI) after catheterization in an otherwise immunocompetent young female patient and a case of UTI in a geriatric patient who was undergoing cancer treatment were reported previously [5,6]. Other infections associated with *Geotrichum *spp.* *include septicemia, renal bezoar (fungal ball), non-invasive sinusitis, and burns wound infections [7-10]. The present report reviews a case funguria with asymptomatic UTI in a 65-year-old male patient who presented to the emergency department with insidious onset of weakness and slurring of speech. 

## Case presentation

**Figure 1:**
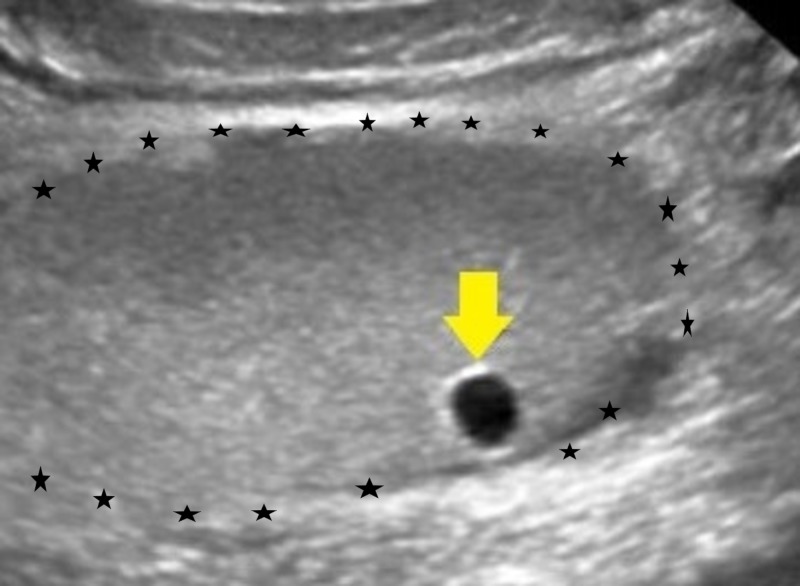
The ultrasound image showing the renal cyst (yellow arrow) in the right kidney (star border)

A 65-year-old male patient presented to the emergency/casualty department attached to the Prathima Institute of Medical Sciences with complaints of weakness of the right upper and lower limbs for the past four days. He also gave a history of slurring speech for four days with a deviation of the mouth towards the left side. The patient was otherwise healthy before the presentation of the symptoms. There was no history of trauma, fever, headache, nausea, and neck stiffness. He was also not a known case of hypertension, diabetes mellitus, tuberculosis, and asthma. On clinical examination, the patient was found to be drowsy and incoherent. The clinical examination revealed a pulse rate of 88 beats/minute and a blood pressure of 150/90 mmHg. The patient was provisionally diagnosed as a case of hemiplegia and was admitted for further evaluation and medical management. On day 2, when the patient complained of turbid and dark-colored urine, an ultrasound abdomen was advised and a midstream urine sample was sent to the clinical microbiology laboratory for further evaluation. The ultrasound abdomen revealed a distended gall bladder with multiple calculi with an average size of 7 mm. Evidence of an 8X8 mm sized cortical cyst in the mid pole of the right kidney with grade 1 renal parenchymal changes was also noted as shown in Figure 1.

**Figure 2:**
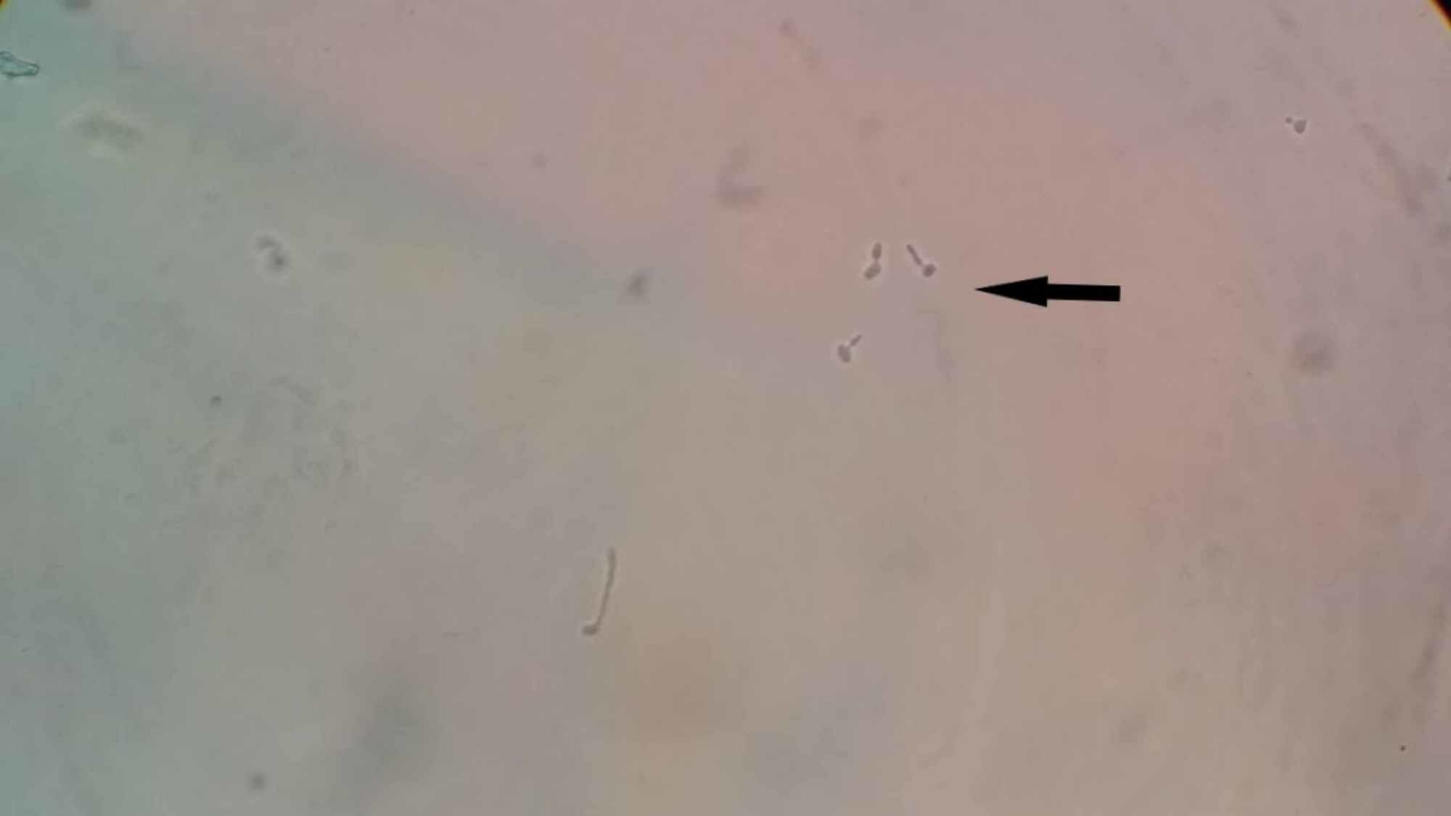
Direct wet mount of the urine showing budding yeast cells (black arrow)

The urine on macroscopic observation was found to be turbid and dark colored. Microscopic examination of the urine showed normal pHand specific gravity, traces of albumin, plenty of red blood cells and pus cells, and occasional epithelial cells. On a simple wet mount of urine, few oval budding yeast cells were noted as shown in Figure 2.

**Figure 3:**
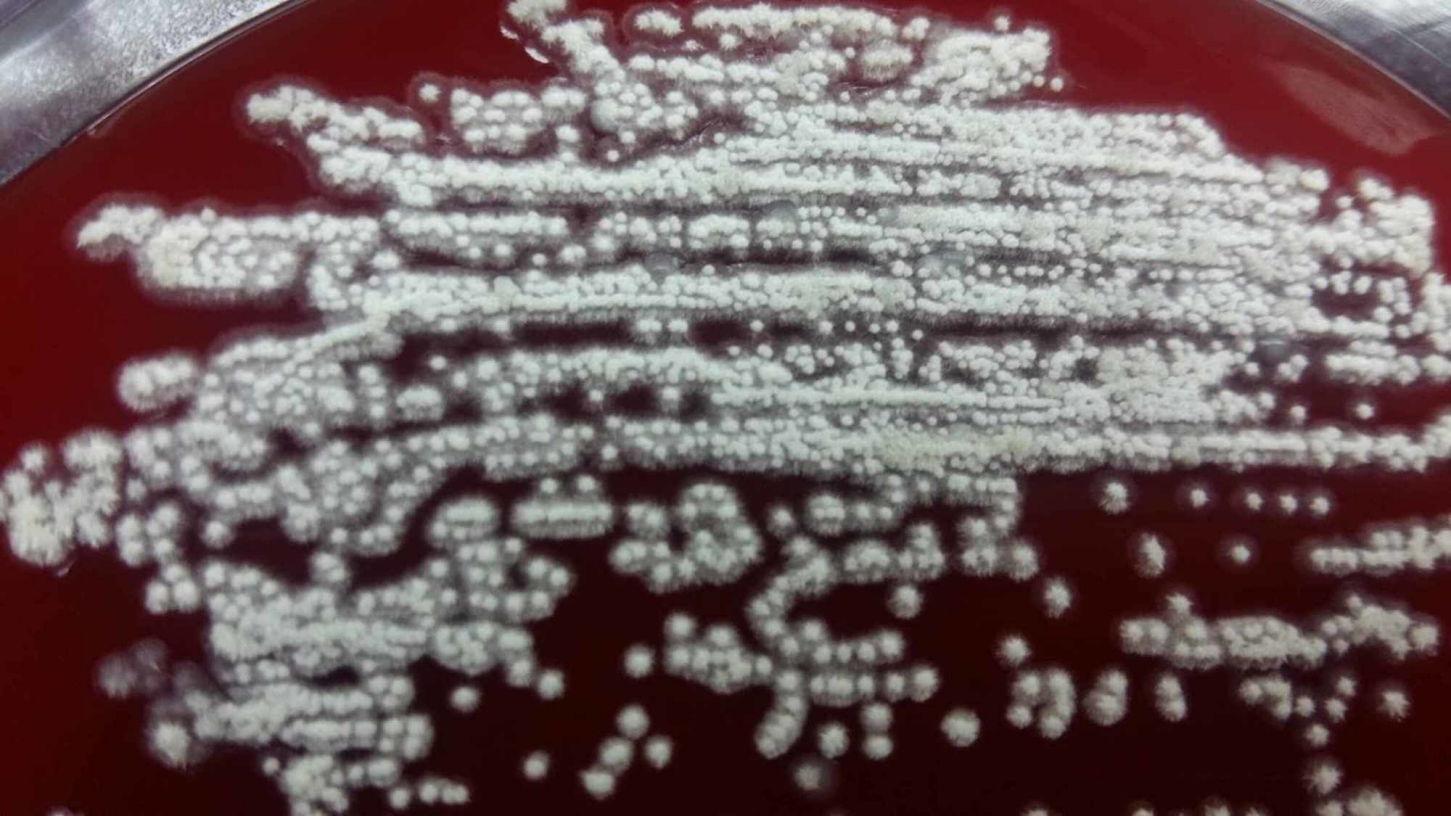
Growth of chalky white-colored stellate colonies on blood agar on the first isolation

The urine was routinely processed for bacterial culture on blood agar and MacConkey's agar. On day 2, the blood agar revealed the growth of chalky white-colored stellate colonies as shown in Figure 3.

**Figure 4:**
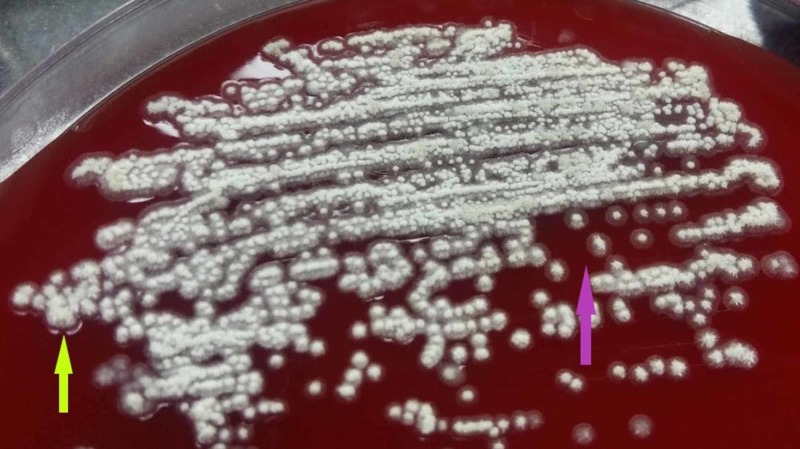
Characteristic pitting colonies (fluorescent green arrow) with grayish hairy bottom/undersurface (fluorescent pink arrow) and the chalky white superficial/central region

The colonies were characteristically revealing pitting (growth of colonies within the agar), with grayish hairy bottom/undersurface and the chalky white superficial/central region as shown in Figure 4.

**Figure 5:**
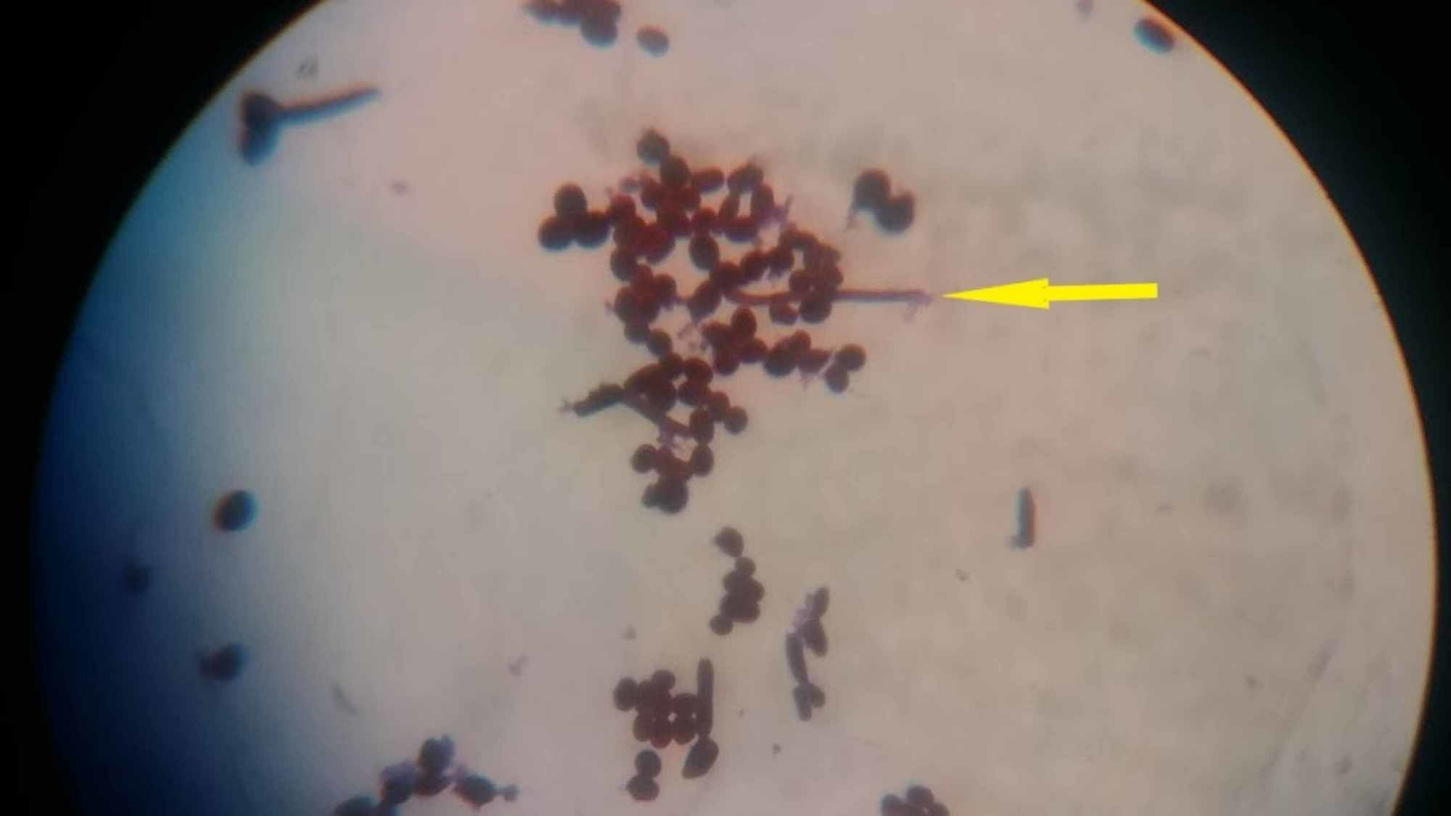
Gram's stain of the growth on blood agar showing Gram-positive oval budding yeast cells with occasional hyphal forms (yellow arrow)

Similar growth was also observed on MacConkey's agar. Gram's stain of the growth on blood agar revealed Gram-positive oval budding yeast cells with occasional hyphal forms as shown in Figure 5.

**Figure 6:**
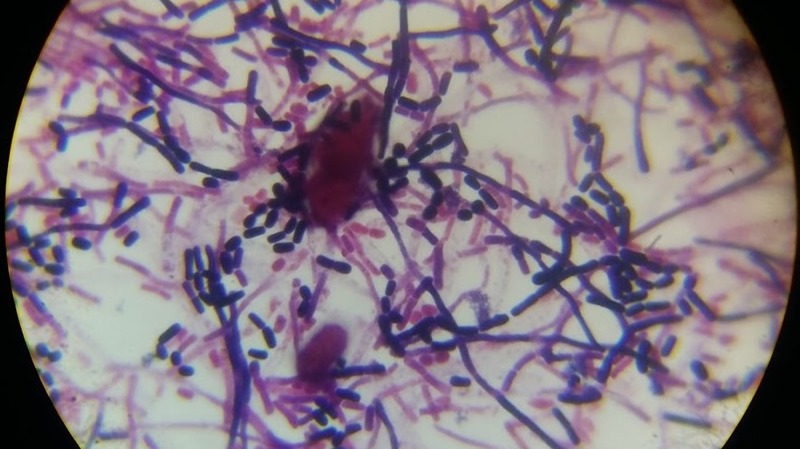
Gram's stain of the colonies showing Gram-positive long filamentous structures that break off to form arthroconidia

After an extended period of incubation, the Gram's stain of the colonies revealed Gram-positive long filamentous structures, which later break off to form arthroconidia as shown in Figure 6.

Antifungal susceptibility testing was performed using the disk diffusion method. The antifungal agents tested included amphotericin B, fluconazole, nystatin, and clotrimazole. The isolated fungus was sensitive to all the antifungal drugs tested. Based on the cultural (stellate colonies with pitting), morphological (presence of arthroconidia), and biochemical (negative urease test) characteristic features, the organism was identified as *Geotrichum *species.

Although the UTI was asymptomatic, considering the age of the patient, and the results of the antifungal susceptibility profile, the patient was treated with amphotericin B (0.6 mg/kg/day)

## Discussion

Fungi, based on their ability to cause infections, are classified as primary pathogens, opportunistic pathogens, and environmental pathogens. The fungi which possess virulence factors and which cause infections even in a healthy individual are called as primary pathogens. *Blastomyces dermatitidis*, *Coccidioides immitis*, *Histoplasma capsulatum*, and others are a few examples of primary pathogens. The opportunistic fungi, which include *Cryptococcus *spp., *Candida *spp., *Torulopsis *spp., *Aspergillus *spp., and others, are either present in the environment as saprophytes or as commensals in humans. These fungi take advantage of the host factors like the immunological deficiency including abnormal phagocytic function, neutropenia, granulocytopenia, metabolic dysfunction, chronic steroid/immunosuppressive therapy, and others to cause infection. The third group of fungi, which comprise the environmental pathogens like *Geotrichum *spp., *Penicillium *spp., *Rhinosporidium *spp., *Sporothrix *spp., and others, causes accidental/occasional/rare human infections, which are worth reporting as rare clinical cases to improve the understanding concerning their virulence, potential predisposing factors, and complications associated with them [11-13]. 

Yeast and yeast-like fungi are a versatile group of microorganisms which consists of several genera like the *Candida*, *Geotrichum (G.)*, *Saccharomyces*, *Torulopsis*, *Rhodotorula*, *Trichosporon*, *Malassezia*, and *Cryptococcus *[13,14]. They are present in the environment as saprophytes, and as commensals in humans/animals. They can cause mild self-limiting infections to severe invasive, and disseminated infections in debilitated, and immunosuppressed individuals. Therefore, most members of this group are recognized as opportunistic fungi. *Geotrichum *is a unique yeast-like fungus, which resents two morphological colony variants, where one type forms glaborous colonies, and resemble other yeasts like the *Candida *spp., and the other variants form fluffy/hairy colonies which resemble the molds/true fungi. Therefore, *Geotrichum *spp. are called imperfect fungi and are placed in the class Fungi Imperfecti [14]. The microscopic appearance of *Geotrichum *differentiates it from the other common yeasts (*Candida*) owing to the presence of long filamentous, branching structures with septa, which later break off to form arthroconidia [1]. 

There are several species of *Geotrichum *that include *G*. *candidum*, *G*. *capitatum*, *G*. *klebahnii*, *G*. *pseudocandidum*, *G*. *citri-aurantii*, *G*. *fermentans*, *G*. *decipiens*, *G*. *restrictum*, *G*. *europaeum*, *G*. *carabidarum*, *G*. *cucujoidarum*, *G*. *histeridarum*,* *and *G. clavatum*. Among these species, *G*. *candidum*, and *G*. *capitatum *are frequently associated with human infections. *Geotrichum *spp. are also associated with granulomatous, and suppurative infections in animals, which include cattle, pigs, dogs, horses, fowl, and others [1]. 

*G*. *candidum *was noted to cause invasive intestinal infection in a patient with hairy cell leukemia. The diagnosis, in this case, was made based on histological and cultural characteristic features. This report proves its invasive capabilities, especially when the host is immunosuppressed [15]. Pulmonary infection with *G*. *capitatum *in a patient with a history of pulmonary tuberculosis was previously reported [16]. This case report highlights the fact that the fungus might take advantage of the debilitating conditions of the host to cause infection. 

The invasiveness of *Geotrichum *spp. and its ability to cause disseminated infection were confirmed by a previous study, which suggested that geotrichosis may in some instances become invasive and fatal. A case reporting multiple abscesses of the kidney caused by *Geotrichum *spp. confirms its potential for tissue invasion [17]. This study had also suggested that despite such reports, it cannot be hypothesized that geotrichosis alone may result in renal failure/pathology because the outcome may be influenced both by the organism and host factors. 

*G*. *candidum *had been isolated from the vitreous fluid in a case of post-cataract endophthalmitis. Despite extended treatment with amphotericin B and voriconazole, the patient suffered enucleation [18]. 

Infection in the immunocompetent individuals, although rare, was infrequently reported. A young male patient was found suffering from a suppurative infection of the metacarpophalangeal joint following a traumatic injury. The joint fluid aspirated from the patient was noted to be seropurulent and had grown *G*. *candidum*. The patient, in this case, was successfully treated with oral ketoconazole for eight weeks [19]. 

The taxonomy of the *Geotrichum *spp. and its teleomorphs (sexual/perfect stages of fungi) has been recently revised. Many species have been renamed based on the phenotypic characteristic features, molecular analysis using the ribosomal deoxyribonucleic acid (DNA) internal transcribed spacer regions, multilocus sequencing, and amplified fragment length polymorphism [20]. The uncertainty over the taxonomy, morphological resemblances to other common yeasts like the *Candida *spp., and the similar clinical and histopathological features could have been the reasons for the underreporting of infections caused by *Geotrichum *spp.

In the present case, the patient was admitted to the casualty/emergency with the symptoms related to stroke. The patient was later found to be suffering from asymptomatic UTI. Urine culture revealed the growth of *Geotrichum *spp., and an ultrasound abdomen showed the presence of a renal cyst with grade I renal parenchymal changes. It is not clear if the renal cyst was the predisposing factor for geotrichosis, or the infection was responsible for cyst formation. The histopathological examination of the renal cyst could have given a clue if the cyst was a cause or an effect of the infection, which unfortunately was not done in the present case. 

## Conclusions

*Geotrichum *spp. are a versatile group of imperfect fungi. Morphologically, they resemble the most common yeast-like fungus, the *Candida *spp. Infections caused by *Geotrichum *spp. are underreported due to their similar clinical and histopathological features with other fungal infections. The available literature signifies the pathogenic potential of *Geotrichum *spp., which not only is present in the environment as a saprophyte but also can cause infections in both immunosuppressed and immunocompetent individuals. In the present case, the patient was over 60-year-old and had asymptomatic UTI. Considering the debilitating conditions of the patient, and the possibility of dissemination in the future, a decision to treat the patient with antifungal therapy was made. 
